# Supplementary material for: Muricauda okinawensis sp. Nov. and Muricauda yonaguniensis sp. Nov., Two Marine Bacteria Isolated from the Sediment Core near Hydrothermal Fields of Southern Okinawa Trough
Source: Microorganisms. 2023 Jun 14;11(6):1580. doi: 10.3390/microorganisms11061580 (PMC10301316; doi:10.3390/microorganisms11061580)
Supplement: Supplementary file 1 [file microorganisms-11-01580-s001.zip › microorganisms-2436427-supplementary.pdf]

Supplementary materials

Muricauda okinawensis sp. Nov. and *Muricauda yonaguniensis* sp. Nov., Two Marine Bacteria Isolated from the Sediment Core Near Hydrothermal Fields of Southern Okinawa Trough

Wenrui Cao [1,*], Xingyu Deng [1,2], Mingyu Jiang [1], Zhigang Zeng [1] and Fengming Chang [1]

[1] Key Laboratory of Marine Geology and Environment, Institute of Oceanology, Chinese Academy of Sciences, Qingdao 266071, China; dengxingyu@qdio.ac.cn (X.D.); myjiang@qdio.ac.cn (M.J.); zgzeng@qdio.ac.cn (Z.Z.); chfm@qdio.ac.cn (F.C.)

[2] College of Earth Science and Engineering, Shandong University of Science and Technology, Qingdao 266590, China

* Correspondence: caoyubeibei@126.com; Tel.: +86-532-82898939

Table S1. ANIb, ANIm and dDDH values between pairs of type strains of *Muricauda* species.

		1	2	3	4	5	6	7	8	9	10	11
**#ANIb value %** **(ANIm value %)**												
1	81s02^{T}	-	81.4 (85.5)	79.6 (83.9)	78.0 (83.0)	80.2 (84.5)	79.7 (84.0)	79.2 (83.5)	79.4 (83.3)	80.8 (84.5)	80.4 (84.9)	79.7 (83.4)
2	334s03^{T}	81.5 (85.6)	-	80.9 (84.3)	78.8 (83.3)	80.9 (84.5)	80.4 (84.0)	80.6 (84.1)	80.7 (83.9)	86.3 (88.4)	81.2 (84.8)	81.0 (84.3)
3	*M. abyssi* W52^{T}	79.4 (83.9)	80.7 (84.3)	-	78.6 (83.4)	91.4 (93.3)	88.0 (89.8)	88.2 (89.8)	79.4 (83.3)	81.3 (85.0)	83.5 (86.6)	80.5 (84.3)
4	*M. aquimarina* SW-63^{T}	78.4 (83.0)	79.3 (83.3)	79.0 (83.4)	-	78.8 (83.1)	78.5 (82.9)	78.7 (83.1)	78.7 (82.7)	79.3 (83.3)	79.5 (83.6)	79.3 (83.2)
5	*M. aurea* BC31-1-A7^{T}	80.1 (84.5)	80.8 (84.5)	91.4 (93.3)	78.3 (83.1)	-	87.6 (89.5)	87.7 (89.4)	79.5 (83.3)	81.4 (85.0)	83.0 (86.1)	79.7 (83.6)
6	*M. brasiliensis* K001^{T}	79.9 (84.0)	80.6 (84.0)	88.6 (89.8)	78.4 (82.9)	88.2 (89.5)	-	95.6 (96.1)	79.4 (83.0)	80.7 (84.2)	83.7 (86.0)	79.9 (83.4)
7	*M. chongwuensis* HICW^{T}	79.6 (83.5)	80.9 (84.1)	88.7 (89.8)	78.7 (83.1)	88.3 (89.4)	95.7 (96.1)	-	79.5 (83.0)	80.8 (84.2)	83.5 (85.9)	80.1 (83.3)
8	*M. lutimaris* SMK-108^{T}	79.9 (83.3)	81.1 (83.9)	79.9 (83.3)	78.7 (82.7)	80.1 (83.3)	79.6 (83.0)	79.7 (83.0)	-	80.8 (83.7)	80.4 (83.8)	82.5 (85.0)
9	*M. oceanensis* 40DY170^{T}	80.8 (84.5)	86.3 (88.4)	81.6 (85.0)	79.0 (83.4)	81.6 (85.0)	80.6 (84.2)	80.7 (84.2)	80.5 (83.7)	-	81.0 (84.7)	81.1 (84.0)
10	*M. oceani* 501str8^{T}	80.1 (84.9)	80.8 (84.9)	83.3 (86.6)	78.9 (83.6)	82.9 (86.1)	82.9 (86.0)	82.9 (85.9)	79.7 (83.8)	80.6 (84.7)	-	80.2 (84.1)

11	*M. ruestringensis* B1^{T}	79.8 (83.5)	81.5 (84.3)	80.9 (84.3)	79.1 (83.2)	80.2 (83.6)	80.0 (83.5)	80.0 (83.3)	82.3 (85.0)	81.3 (84.0)	80.8 (84.1)	-
**#dDDH value %**												
1	81s02^{T}	-										
2	334s03^{T}	25.4	-									
3	*M. abyssi* W52^{T}	23.0	24.6	-								
4	*M. aquimarina* SW-63^{T}	21.5	22.5	22.1	-							
5	*M. aurea* BC31-1-A7^{T}	23.8	24.7	49.4	21.9	-						
6	*M. brasiliensis* K001^{T}	23.3	24.0	37.4	21.6	36.5	-					
7	*M. chongwuensis* HICW^{T}	22.8	24.2	37.3	21.8	36.3	67.0	-				
8	*M. lutimaris* SMK-108^{T}	22.8	24.7	23.1	21.8	23.2	22.7	22.8	-			
9	*M. oceanensis* 40DY170^{T}	24.5	33.9	25.3	22.4	25.4	24.1	24.2	24.2	-		
10	*M. oceani* 501str8^{T}	24.1	25.1	28.8	22.8	27.9	27.9	27.7	23.7	25.0	-	
11	*M. ruestringensis* B1^{T}	23.1	25.5	24.7	22.6	23.7	23.4	23.5	26.5	25.0	24.4	-

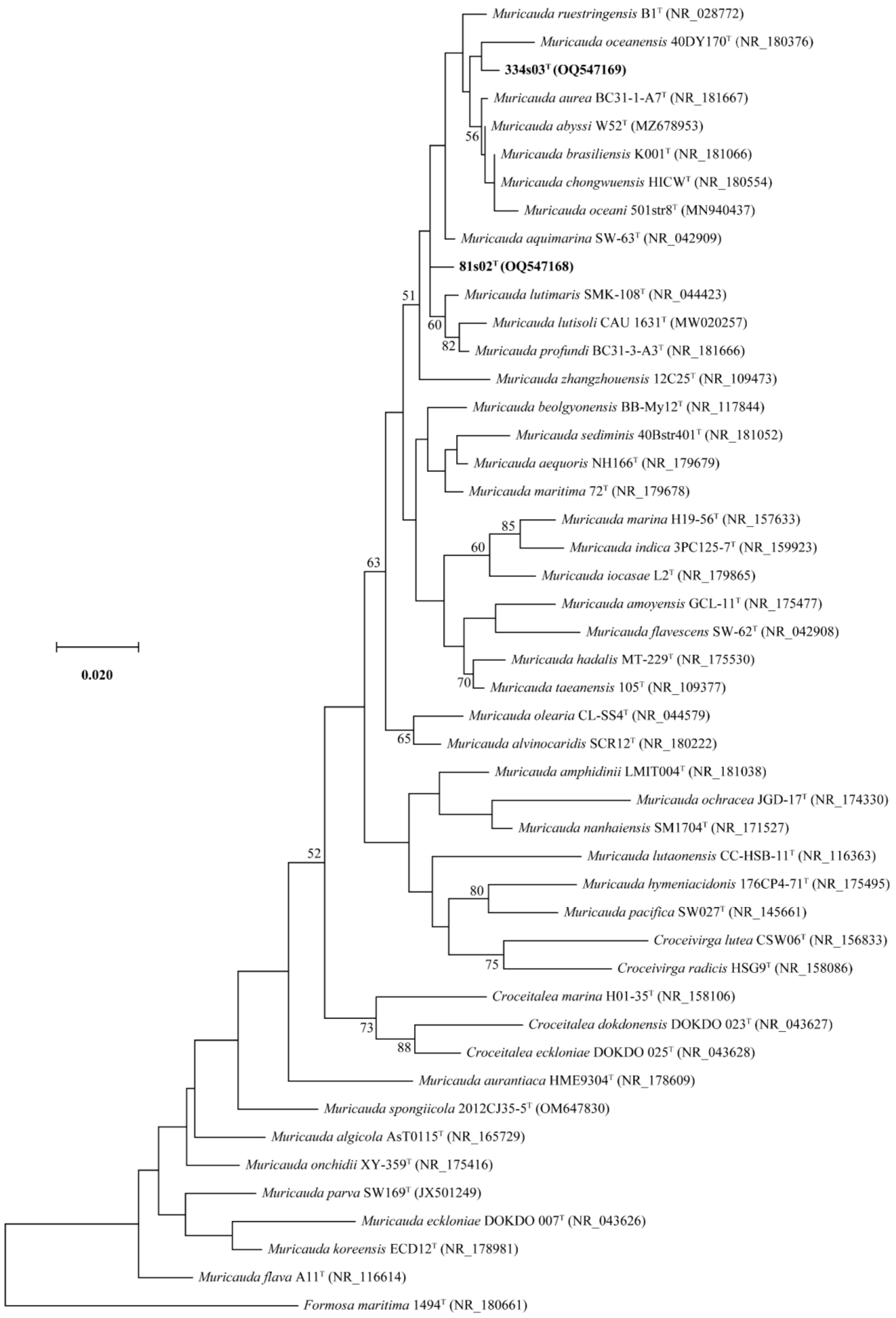

Figure S1. Phylogenetic tree, based on the 16S rRNA gene sequences using the maximum-likelihood algorithm showing the position of strains 81s02^{T} and 334s03^{T}. GenBank accession numbers used are given in the parentheses. Bootstrap values higher than 50 % are indicated at branch nodes. *Formosa maritima* 1494^{T} was used as outgroup. Bar, 0.02 substitutions per nucleotide position.

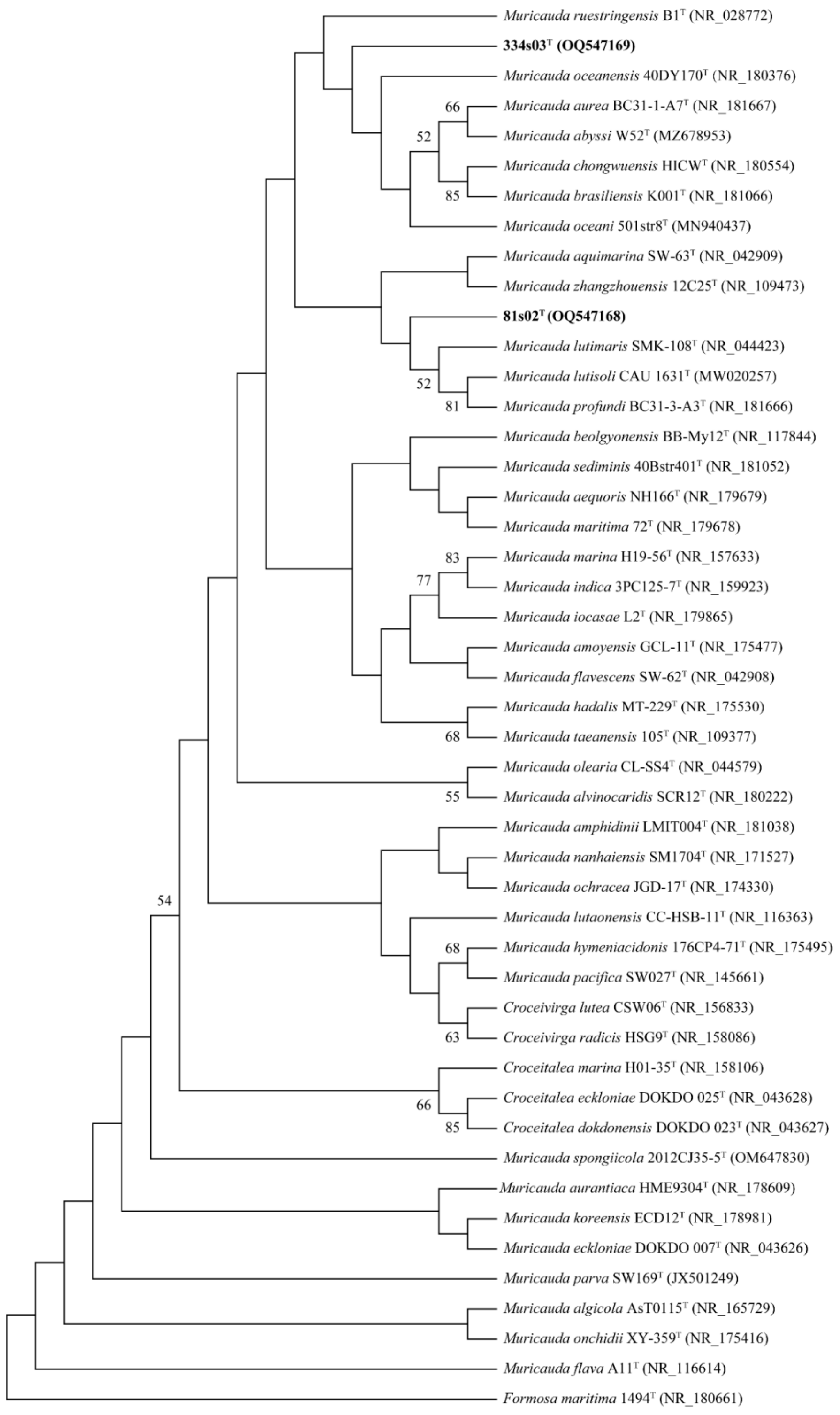

Figure S2. Phylogenetic tree, based on the 16S rRNA gene sequences using the maximum-parsimony algorithm showing the position of strains 81s02^T and 334s03^T. GenBank accession numbers used are given in the parentheses. Bootstrap values higher than 50 % are indicated at branch nodes.

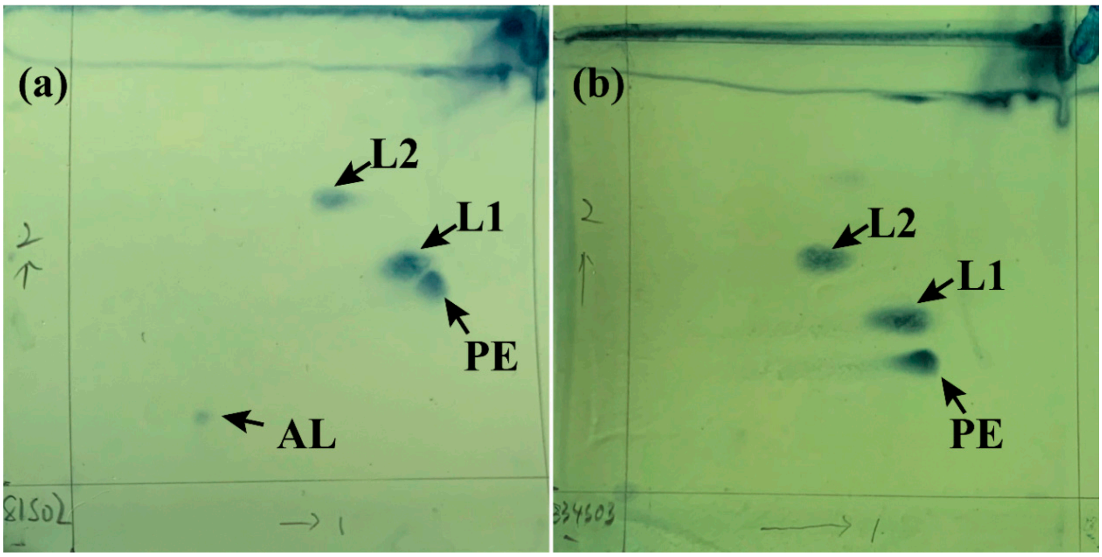

Figure S3. Two-dimensional thin-layer chromatogram of polar lipids. (a) 81s02^{T}; (b) 334s03^{T}. PE, phosphatidylethanolamine; AL, unidentified aminolipid; L 1-2, unidentified lipids.

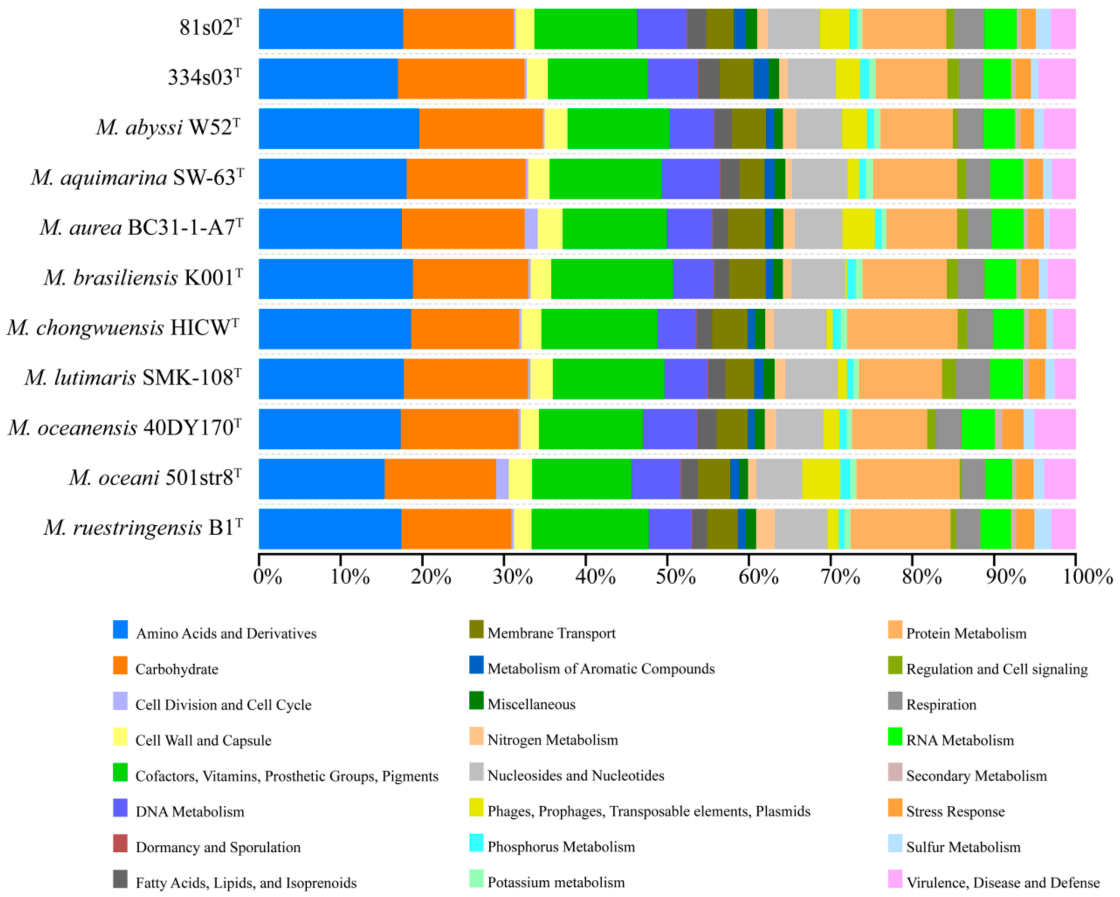

Figure S4. Metabolic features related to functional categories of 11 *Muricauda* strains. The encoding gene involved in the categories of "Photosynthesis", "Iron acquisition and metabolism" and "Motility and Chemotaxis" was not annotated.

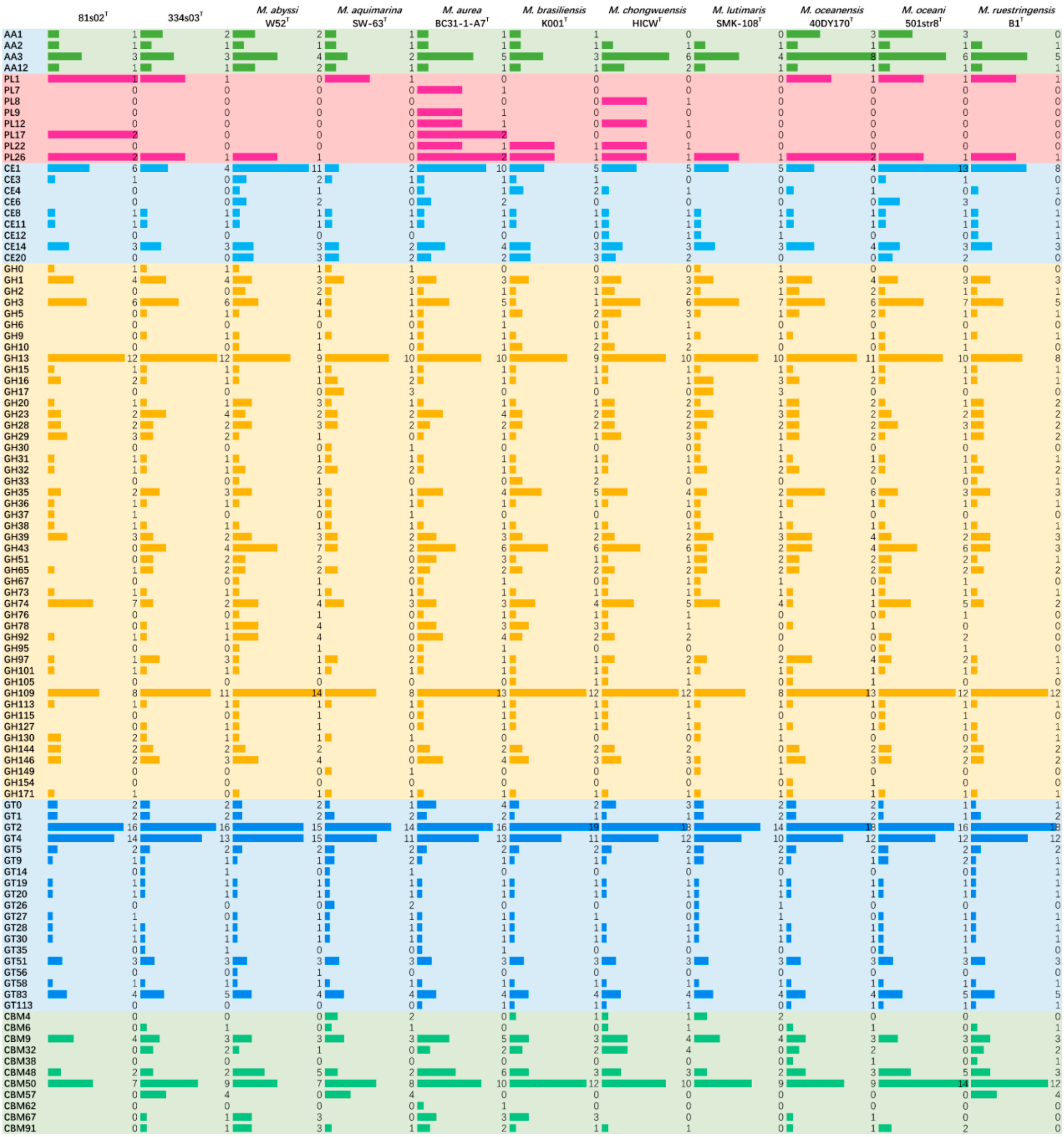

Figure S5. Genes putatively coding for carbon metabolism among different *Muricauda* species based on the CAZy database. AAs, redox enzymes with auxiliary activities; PLs, polysaccharide lyases; CEs, carbohydrate esterases; GHs, glycoside hydrolases; GTs, glycosyltransferases; CBMs, carbohydrate-binding modules.
